# Tumor location matters, next generation sequencing mutation profiling of left-sided, rectal, and right-sided colorectal tumors in 552 patients

**DOI:** 10.1038/s41598-024-55139-w

**Published:** 2024-02-26

**Authors:** Izabela Ciepiela, Magdalena Szczepaniak, Przemysław Ciepiela, Kinga Hińcza-Nowak, Janusz Kopczyński, Paweł Macek, Kamila Kubicka, Magdalena Chrapek, Magdalena Tyka, Stanisław Góźdź, Artur Kowalik

**Affiliations:** 1 Radiotherapy Department, Holy Cross Cancer Centre, 25-734 Kielce, Poland; 2 Department of Molecular Diagnostics, Holy Cross Cancer Centre, 25-734 Kielce, Poland; 3 Surgical Oncology Department, Holy Cross Cancer Centre, 25-734 Kielce, Poland; 4 Endocrinology Clinic, Holy Cross Cancer Centre, 25-734 Kielce, Poland; 5 Surgical Pathology, Holy Cross Cancer Centre, 25-734 Kielce, Poland; 6 Collegium Medicum, Jan Kochanowski University, 25-319 Kielce, Poland; 7 Department of Epidemiology and Cancer Control, Holy Cross Cancer Centre, 25-734 Kielce, Poland; 8 Department of Mathematics, Faculty of Natural Sciences, Jan Kochanowski University, 25-406 Kielce, Poland; 9 Clinical Oncology Clinic, Holy Cross Cancer Centre, 25-734 Kielce, Poland; 10 Division of Medical Biology, Institute of Biology, Jan Kochanowski University, 25-406 Kielce, Poland

**Keywords:** Cancer genetics, Cancer genomics, Tumour biomarkers, Cancer, Gastrointestinal cancer, Colorectal cancer, Colon cancer, Rectal cancer

## Abstract

Despite the introduction of new molecular classifications, advanced colorectal cancer (CRC) is treated with chemotherapy supplemented with anti-EGFR and anti-VEGF targeted therapy. In this study, 552 CRC cases with different primary tumor locations (250 left side, 190 rectum, and 112 right side) were retrospectively analyzed by next generation sequencing for mutations in 50 genes. The most frequently mutated genes were *TP53* in left-sided tumors compared to right-sided tumors and *BRAF* in right-sided tumors compared to left-sided tumors. Mutations in *KRAS*, *NRAS*, and *BRAF* were not detected in 45% of patients with left-sided tumors and in 28.6% of patients with right-sided tumors. Liver metastases were more common in patients with left-sided tumors. Tumors on the right side were larger at diagnosis and had a higher grade (G3) than tumors on the left. Rectal tumors exhibit distinctive biological characteristics when compared to left-sided tumors, including a higher absence rate of *KRAS, NRAS*, and *BRAF* mutations (47.4% in rectal versus 42.8% in left-sided tumors). These rectal tumors are also unique in their primary metastasis site, which is predominantly the lungs, and they have varying mutation rates, particularly in genes such as *BRAF*, *FBXW7*, and *TP53*, that distinguish them from tumors found in other locations. Primary tumor location has implications for the potential treatment of CRC with anti-EGFR therapy.

## Introduction

Colorectal cancer (CRC) is one of the most common causes of cancer-related death in highly developed countries. In 2020, approximately 1.9 million new cases of CRC were diagnosed worldwide, and more than 0.9 million CRC-related deaths were reported^1^. In the same year, there were approximately 148,000 new cases of CRC in the United States, and approximately 53,000 patients died from this disease^2^. In Europe, there were approximately 500,000 new cases of CRC in 2018 and 243,000 deaths from this disease^3^.

CRC develops gradually from adenoma to carcinoma (adenoma to carcinoma sequence). More than 30 years ago, Fearon and Vogelstein proposed a stepwise model of CRC carcinogenesis^4^. Subsequent molecular findings confirmed the model proposed by Fearon and Vogelstein. Morphological changes are accompanied by molecular changes. In the "classical" model of adenoma to carcinoma [chromosomal instability and microsatellite instability (MSI) in Lynch syndrome], carcinogenesis begins with mutations in *APC*, followed by a sequence of mutations in the *KRAS/NRAS* genes and in *SMAD4*, and finally mutations in *TP53*. In an alternative pathway, a subset of CRCs arise from serrated polyps as precursor lesions that progress to cancer (CIMP and sporadic MSI). The earliest mutations arise in the *CTNNB1* gene, progressing through mutations in the *KRAS*/*BRAF* genes and *PIK3CA*, and finally mutations in *TGFBR1*^5^. Large-scale gene expression analyses led to the classification of CRC into four consensus molecular subtypes (CMS1–4): MSI immune [CMS1], canonical [CMS2], metabolic [CMS3], and mesenchymal [CMS4]^6^.

Despite the introduction of new molecular subdivisions (CMS1–4), advanced CRC is treated systemically with chemotherapy (e.g., FOLFOX) supplemented with anti-EGFR and anti-VEGF targeted therapy^7^. Additionally, patients are stratified for anti-EGFR therapy according to the presence of mutations in the *KRAS*, *NRAS*, and *BRAF* genes determined by molecular diagnostics. Targeted anti-EGFR therapy only benefits patients without these mutations^8^. Encouraging therapeutic results have been obtained using anti-PD1 immune checkpoint inhibitors (e.g., nivolumab) for the treatment of advanced CRC. Patients with disseminated CRC who qualify for immunotherapy undergo immunohistochemical evaluation of mismatch repair (MMR) or assessment of the genetic presence of MSI^9^. Although MMR/MSI are detected in only 15% of CRCs, clinical trials are underway to evaluate the possible use of anti-PD1 immunotherapy in MMR-proficient CRC^10^.

CRC is not a single type of cancer, and its pathogenesis depends on the anatomical location of the tumor, including differences between the right and left sides of the colon. Tumors in the proximal (right) and distal (left) parts of the colon show different molecular and histological features. Mutations in the DNA MMR pathway are commonly observed in right-sided tumors, and these tumors generally have a flat histology. In left-sided tumors, mutations related to the chromosomal instability pathway, such as *KRAS*, *APC*, *PIK3CA*, and p53 mutations, are common, and these tumors have a polyp-like morphology^11^. In general, patients with left-sided tumors have a better prognosis than those with right-sided tumors^11–13^. However, right-sided tumors that are detected in early stages (I and II) have a better prognosis, whereas left-sided tumors have a better prognosis in late stages (stages III and IV)^14^. Patients with tumors located on the left side typically benefit from adjuvant chemotherapy, such as 5-fluorouracil (5-FU)-based treatment, and therapies targeting EGFR. Patients with tumors located on the right side do not respond well to conventional chemotherapies and do not benefit from anti-EGFR therapy as first-line treatment for generalized disease; this may be attributed to the differences in embryologic origin between right- and left-sided tumors^11,12^. In cancers located on the right side, immunotherapy has shown promising results because these tumors have a high tumor mutation burden^15^. Therefore, the stratification of patients for targeted therapy and the design of effective treatment strategies require consideration of the location of the primary tumor i.e., left side or right side.

In this study, mutations in 50 genes were examined by next generation sequencing (NGS) in 552 tissue samples of primary CRC tumors, and the results were retrospectively analyzed. The aim of the study was to analyze the clinical and mutational data according to primary tumor locations. First left-sided vs. right-sided tumors were compared. Then we excluded rectum tumors from left-sided tumors and compared clinical and genetic data according to three locations left-sided tumor vs. rectum vs. right-sided tumor.

## Results

Of 552 CRC cases analyzed, 440 tumors were located on the left side (including 190 cases of cancer located in the rectum), and 112 tumors were located on the right side (Table 1).

**Table 1:** Clinicopathological characteristics of the 552 colorectal cancer cases studied.

Characteristics	N = 552
Age	
N	552
Mean (SD)	65 (10)
Median (IQR)	66 (59, 71)
Range	27, 90
Sex	
Man	343 (62%)
Woman	209 (38%)
Tumor_location.v1	
Left	250 (45%)
Rectal	190 (34%)
Right	112 (20%)
Tumor_location.v2	
Left	440 (80%)
Right	112 (20%)
Clinical_stage	
0	3 (0.5%)
I	27 (4.9%)
II	52 (9.4%)
III	82 (15%)
IV	388 (70%)
HP	
Adenoca	21 (3.8%)
Adenoca G1	84 (15%)
Adenoca G2	392 (71%)
Adenoca G3	48 (8.7%)
Adenoca Gx	1 (0.2%)
Goblet cell carcinoid	1 (0.2%)
Medullary carcinoma	1 (0.2%)
NA	3 (0.5%)
Neuroendocrine carcinoma	1 (0.2%)

Adenocar Gx—adenocarcinoma without data about grading not available, *HP* histopathology type, *NA* histopathology type not available.

**Figure 1:**
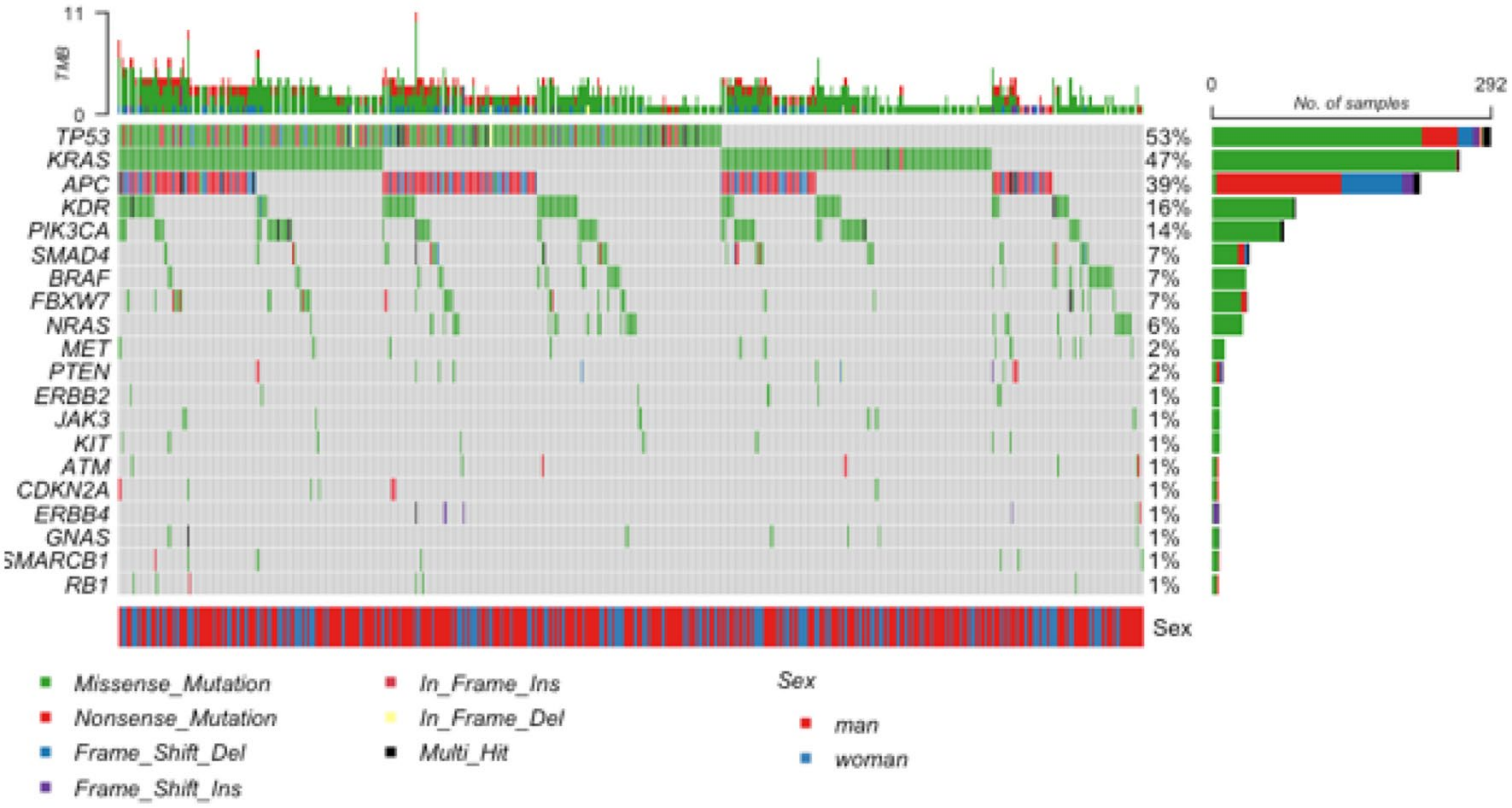
Oncoplot depicting the most recurrent (> 1%) genomic alterations in 552 colorectal cancer cases. Each column represents a tumor, and the bar graph (tumor mutation burden -TMB) at the top shows the number/distribution of mutations detected per sample. The Oncoprint rows show the changes for each gene. The summarized gender information for each case is shown at the bottom of the graph. The bar graph on the right side of the panel shows the number and distribution of mutations for each gene. Mutation types and gender are color-coded according to the legend.

At least one mutation was detected in 90.6% (500/552) of the studied cases. Most of the mutations detected were single-nucleotide missense mutations. A total of 1223 mutations were detected in the 500 cases. On average, 2.2 mutations were detected per case (range: 0–11; sd: 1.38; median: 2; IQR: 1–3). In 9.4% (52/552) of cases, no mutation was detected in 50 of the genes tested (Fig. 1).

In the entire study group of 552 cases, the most frequent mutations were in the *TP53* gene (52% of the samples), followed by *KRAS* (47%) and *APC* (39%) (Fig. 1). Overall, mutations in each of the following eleven genes (*TP53*, *KRAS*, *APC*, *KDR*, *PIK3CA*, *SMAD4*, *BRAF*, *FBXW7*, *NRAS*, *MET*, and *PTEN*) were most often (≥ 14 mutations) detected in our cohort. Mutations in other genes (*SMARCB1*, *ERBB2*, *JAK3*, *KIT*, *ATM*, *CDKN2A*, *GNAS*, *EGFR*, *ERBB4*, *RB1*, *CTNNB1*, *RET*, *STK11*, *FGFR3*, *FLT3*, *VHL*, *AKT1*, *FGFR1*, *IDH1*, *IDH2*, *ABL1*, *CDH1*, *EZH2*, *GNAQ*, *JAK2*, *NOTCH1*, and *PTPN11*) were present at lower frequencies. No mutations were detected in 12 genes (*ALK*, *CSF1R*, *FGFR2*, *GNA11*, *HNF1A*, *HRAS*, *MPL*, *NPM1*, *PDGR*, *SMO*, *SRC*, and *MLH1*) (Fig. 1).

### Mutations in the *KRAS* gene

Mutations in the *KRAS* gene were detected in 261 (47%) cases (Fig. 1). The mutations p.(Gly12Asp), p.(Gly12Val), and p.(Gly13Asp) represented 66.7% of all *KRAS* gene mutations detected. Additionally, 19 p.(Gly12Cys) mutations (7.3%; 19/261) were detected. However, in the whole group (all 552 cases included in the study), the *KRAS* p.(Gly12Cys) mutation was detected in 3.4% (19/552) of cases (Supplementary Table 1).

### Mutations in the *NRAS* gene

Mutations in the *NRAS* gene were present in 32 (6%) of the 552 CRC cases studied (Fig. 1). The most abundant *NRAS* mutations were p.(Gln61Lys), p.(Gly12Asp), and p.(Gln61Arg), which were detected in 23 (71.9%) of the 32 cases (Supplementary Table 2).

### Mutations in the *BRAF* gene

Mutations in the *BRAF* gene were detected in 36 (7%) of the 552 cases studied (Fig. 1). The p.(Val600Glu) mutation was detected in 22 (61%) of the 36 cases. In the remaining 14 cases, the mutations involved other codons in the *BRAF* gene. Among these 14 cases, 4 also had mutations in *KRAS*, and one had a mutation in *NRAS*. None of the cases with the p.(Val600Glu) mutation had mutations in the *KRAS* or *NRAS* gene (Supplementary Table 3).

### Mutations in the *PIK3CA* gene

Mutations in the *PIK3CA* gene were detected in 76 (14%) of the 552 cases (Fig. 1). Three mutations (p.(Glu545Lys), p.(Glu542Lys), and p.(His1047Arg)) were present in more than half (57.3%) of the 76 cases with *PIK3CA* mutations; these mutations are in exons 9 and 20 (Supplementary Table 4). Seventeen (3%, 17/552) cases without mutations in *KRAS*, *NRAS*, and *BRAF* genes had mutations in *PIK3CA*. Eight mutations (4 × p.(Glu542Lys), 4 × p.(Glu545Lys)) were in exon 9, and five (0.9%) mutations (3 × p.(His1047Arg), 1 × p.(Tyr1021Phe), and one double p.(Arg88Gln)&p.(Asn1044Ser) were in exon 20 (Supplementary Table 5). Seven (1.2%) of the analyzed cases had mutations in the *PTEN* suppressor gene in addition to *PIK3CA* mutations.

### Mutations in the *ERBB2* (HER2) gene

Mutations in the *ERBB2* gene (p.(Leu755Ser) in two, p.(Arg784His) in two, and one case each of p.His295AspfsTer16, p.(Val762Leu), p.(Val777Leu), and p.(Val842Ile)) were detected in 8 (1.4%) of the 552 CRC cases analyzed. Six mutations were present in tumors located on the left side (including three cases in the rectum), and two mutations were in tumors on the right side.

**Figure 2:**
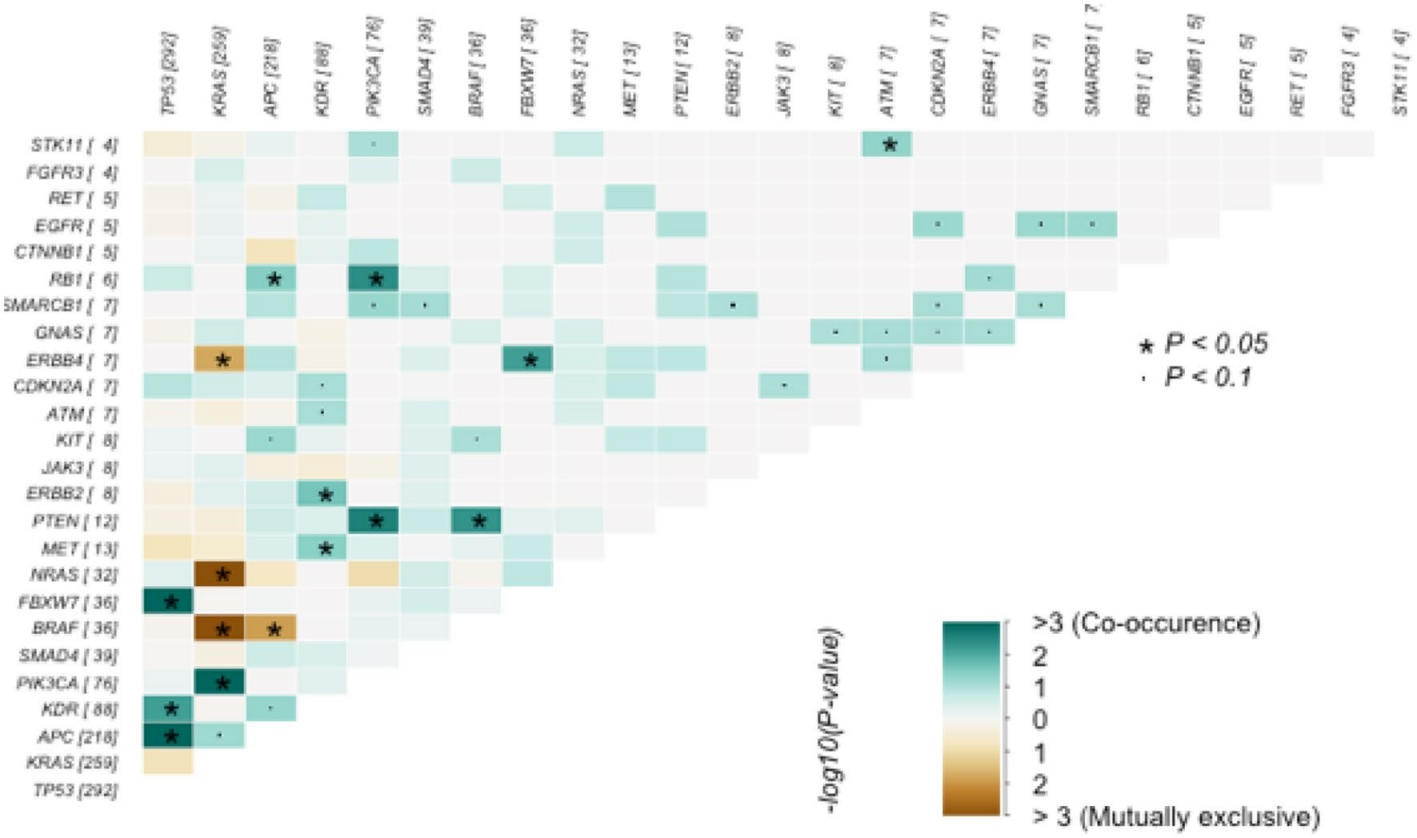
Exclusive/co-occurrence event analysis of the top 25 mutated genes (p < 0.05, pairwise Fisher’s Exact test).

The relations between the mutations were analyzed in the group of 552 cases (Fig. 2). We detected the following statistically significant correlations (p < 0.05, pairwise Fisher's Exact test): mutations in *TP53* co-occurred with mutations in *FBWX7*, *KDR*, and *APC*. Mutations in the *KRAS* gene co-occurred with mutations in *PIK3CA* and were mutually exclusive with mutations in *NRAS* and *BRAF*. Mutations in the *APC* gene co-occurred with mutations in the *RB1* gene and were mutually exclusive with mutations in the *BRAF* gene. Mutations in the *KDR* gene co-occurred with mutations in *ERBB2* and *MET*. Mutations in the *PIK3CA* oncogene co-occurred with mutations in the tumor suppressor genes *RB1* and *PTEN*. By contrast, mutations in another suppressor gene, *FBXW7*, co-occurred with mutations in the *ERBB4* oncogene. Similarly, mutations in the *BRAF* oncogene co-occurred with mutations in the *PTEN* suppressor gene. Mutations in the *ATM* gene co-occurred with mutations in the *STK11* gene. No statistically significant interactions were detected in the remaining pairs of mutated genes (Fig. 2).

Analysis of mutation frequency in the 552 cases at the pathway level showed activation of the following pathways: RTK-RAS, PI3K, TP53, Cell_Cycle, WNT, NOTCH, and TGF-Beta. The most frequently activated pathways were RTK-RAS, PI3K, and TP53. Supplementary Fig. 1.

### Analysis based on primary tumor location

#### Left vs. right tumor location

Mutation frequency was compared between left- and right-sided tumors (Table 2). Correlation analysis showed that men were more commonly affected than women, especially regarding tumors located on the left side (p = 0.006) (Table 2). *BRAF* mutations were threefold more common (15.2 vs. 4.3%, p < 0.0001) in patients with right-sided tumors than in those with left-sided tumors. Restricting the analysis to the *BRAF* p.(Val600Glu) mutation showed that it was > fourfold more common in the right than in the left side of the colon (10.7 vs. 2.3%, p = 0.0003).

**Table 2:** Correlation of clinical features with detected gene mutations according to primary tumor location (left side and right side).

	Left (N = 440)	Right (N = 112)	Total (N = 552)	p-value
Sex				0.0060
Woman	154 (35.0%)	55 (49.1%)	209 (37.9%)	
Man	286 (65.0%)	57 (50.9%)	343 (62.1%)	
Age (years)				0.6097
< = 49	38 (8.6%)	8 (7.1%)	46 (8.3%)	
> = 50	402 (91.4%)	104 (92.9%)	506 (91.7%)	
Age (years)				0.3368
< = 64	199 (45.2%)	45 (40.2%)	244 (44.2%)	
> = 65	241 (54.8%)	67 (59.8%)	308 (55.8%)	
*BRAF*_all				< 0.0001
No	421 (95.7%)	95 (84.8%)	516 (93.5%)	
Yes	19 (4.3%)	17 (15.2%)	36 (6.5%)	
*BRAF*_(only p.(Val600Glu))				0.0003
No	430 (97.7%)	100 (89.3%)	530 (96.0%)	
Yes	10 (2.3%)	12 (10.7%)	22 (4.0%)	
*KRAS*_all				0.0882
No	240 (54.5%)	51 (45.5%)	291 (52.7%)	
Yes	200 (45.5%)	61 (54.5%)	261 (47.3%)	
*NRAS*				0.4990
No	413 (93.9%)	107 (95.5%)	520 (94.2%)	
Yes	27 (6.1%)	5 (4.5%)	32 (5.8%)	
Mutations				0.0002
Without mutation BKN	197 (44.8%)	32 (28.6%)	229 (41.5%)	
* BRAF*	17 (3.9%)	14 (12.5%)	31 (5.6%)	
* KRAS*	199 (45.2%)	61 (54.5%)	260 (47.1%)	
* NRAS*	27 (6.1%)	5 (4.5%)	32 (5.8%)	
Tumor_recurrence				0.1781
N-Miss	1	0	1	
No	398 (90.7%)	106 (94.6%)	504 (91.5%)	
Yes	41 (9.3%)	6 (5.4%)	47 (8.5%)	
ICD				NA
C18	179 (40.7%)	112 (100.0%)	291 (52.7%)	
C19	71 (16.1%)	0 (0.0%)	71 (12.9%)	
C20	190 (43.2%)	0 (0.0%)	190 (34.4%)	
Liver metastases				0.0411
No	200 (45.5%)	63 (56.2%)	263 (47.6%)	
Yes	240 (54.5%)	49 (43.8%)	289 (52.4%)	
Lung metastases				0.3240
No	330 (75.0%)	89 (79.5%)	419 (75.9%)	
Yes	110 (25.0%)	23 (20.5%)	133 (24.1%)	
Peritoneum metastases				0.1095
No	406 (92.3%)	98 (87.5%)	504 (91.3%)	
Yes	34 (7.7%)	14 (12.5%)	48 (8.7%)	
Grade				0.0001
N-Miss	23	5	28	
Adenoca G1	69 (16.5%)	15 (14.0%)	84 (16.0%)	
Adenoca G2	321 (77.0%)	71 (66.4%)	392 (74.8%)	
Adenoca G3	27 (6.5%)	21 (19.6%)	48 (9.2%)	
T				0.0277
N-Miss	145	26	171	
T1	10 (3.4%)	0 (0.0%)	10 (2.6%)	
T2	32 (10.8%)	7 (8.1%)	39 (10.2%)	
T3	183 (62.0%)	45 (52.3%)	228 (59.8%)	
T4	65 (22.0%)	33 (38.4%)	98 (25.7%)	
Tis	2 (0.7%)	1 (1.2%)	3 (0.8%)	
Tx	3 (1.0%)	0 (0.0%)	3 (0.8%)	
N				0.0640
N-Miss	145	26	171	
N0	106 (35.9%)	26 (30.2%)	132 (34.6%)	
N1	111 (37.6%)	25 (29.1%)	136 (35.7%)	
N2	73 (24.7%)	34 (39.5%)	107 (28.1%)	
Nx	5 (1.7%)	1 (1.2%)	6 (1.6%)	

*N-Miss* number of cases without information, *ICD* International Classification of Diseases, *T* tumor, *N* node, *BKN BRAF, KRAS, NRAS*, *NA* not applicable.

Mutations in *KRAS*, *NRAS*, and *BRAF* were not detected in 28.6% of patients with right-sided tumors, whereas 45% of patients with left-sided tumors had no mutations in these genes (p = 0.0002). By contrast, liver metastases were more common in patients with tumors located on the left side (p = 0.0411). Tumors on the right side were more likely to show a large size at diagnosis than those located on the left side (p = 0.0277). Tumors located on the right side were threefold more likely (19.6 vs. 6.5%) to be of the highest histological malignancy grade (G3) (p = 0.0001). Other characteristics analyzed were age (< 49 vs. > 50 and < 64 vs. > 65), *KRAS* and *NRAS* status, presence of recurrence, lung and peritoneal metastases, and T and N stage, which showed no statistically significant differences between cases with left- and right-sided tumor localization (Table 2).

Mutations in *TP53*, *FBXW7*, *SMAD4*, and *NRAS* were more frequent in tumors located on the left side of the colon, whereas mutations in *KRAS*, *PIK3CA*, *BRAF*, *MET*, and *PTEN* were more frequent in tumors located on the right side of the colon. However, statistically significant differences in the frequency of mutations between the two sided locations were only observed for *TP53* (55.9 vs. 41.1%, p = 0.005) and mentioned above *BRAF* (15.2 vs. 4.3%, p < 0.0001). The remaining genes studied showed no statistically significant differences between the two locations, and mutations in the *APC* gene occurred with similar frequency in the two locations (Supplementary Table 6).

**Figure 3:**
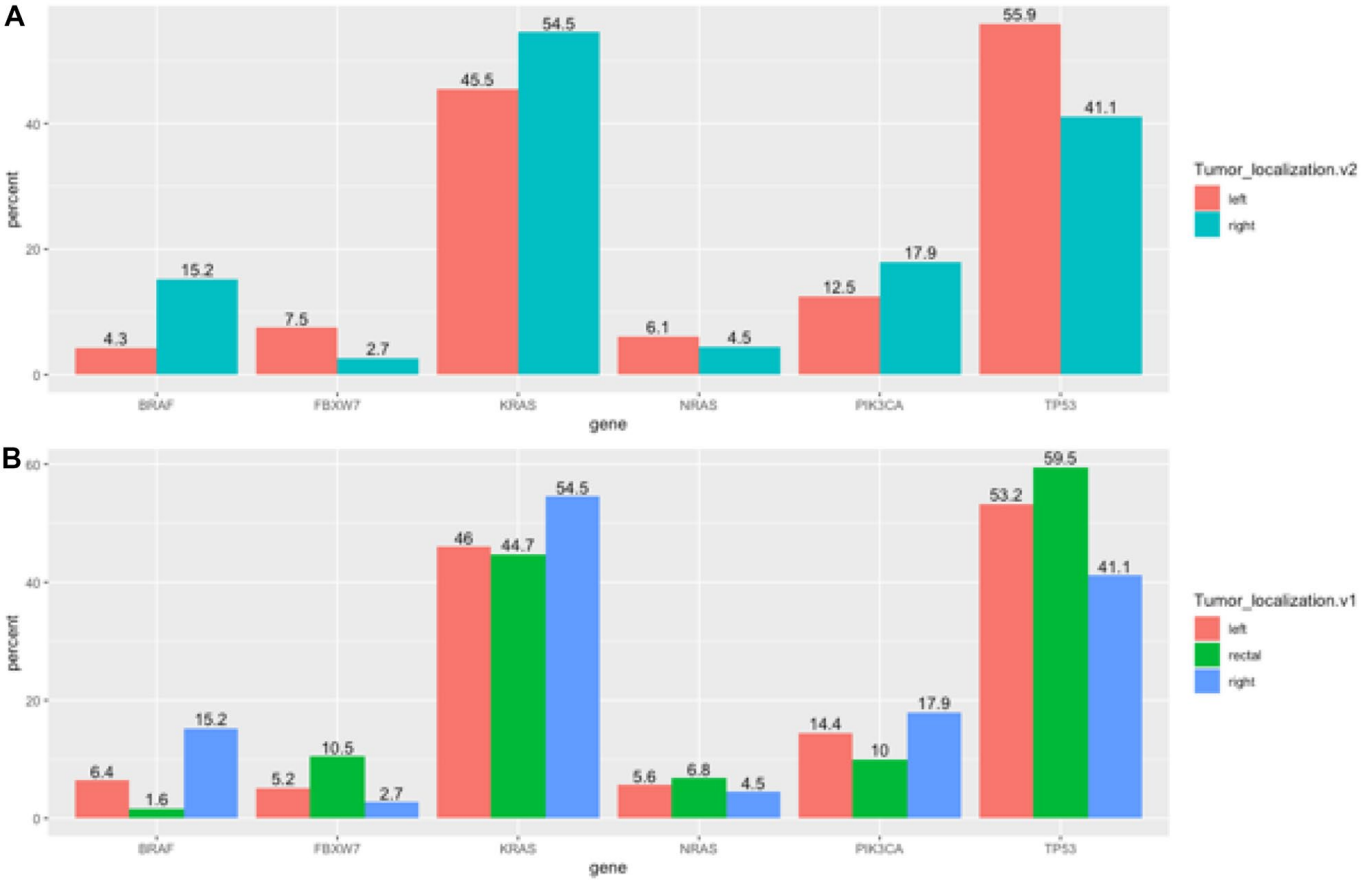
Frequency of mutations detected in the selected genes (*BRAF*, *FBXW7*, *KRAS*, *NRAS*, *PIK3CA*, and *TP53*) according to primary tumor location. (**A)** (right side = 112 cases vs. left side = 440 cases): *BRAF* and *TP53* show statistically significant differences. (**B)** (right side = 112 cases, left side = 250 cases, and rectum = 190 cases): *BRAF*, *FBWX7*, and *TP53* show statistically significant differences.

The findings of the study suggest that left-sided tumors are predominantly observed in males, associated with mutations in the *TP53* gene and an increased likelihood of liver metastases (Fig. 3A, Table 3). Conversely, right-sided cancers are linked to alterations in the *BRAF* gene (Fig. 3A), typically present as larger tumors, and often exhibit a more advanced stage of disease upon initial diagnosis (Table 3).

**Table 3:** Characteristics of right-sided versus left-sided tumors.

	Right-sided	Left-sided
Mutated genes	KRAS, PIK3CA, BRAF*, MET, PTEN	TP53*, FBWX7, SMAD4, NRAS
Sex	Women < Men	Women < Men*
*KRAS, NRAS, BRAF*; WT	28.6%*	45%*
Metastases	Peritoneum	Liver*, Lung
Tumor diameter	Bigger*	Smaller*
Grade	G3*	G2*

*Statistically significant, p < 0.05.

#### Left side vs. right side vs. rectum

The study cohort was divided into three groups according to tumor location as follows: left, right, and rectum (excluding rectum-localized tumors from the group of left-sided tumors) (Table 4). In tumors located in the rectum, the most common mutations were in the *TP53*, *FBXW7*, and *NRAS* genes. In tumors located on the left side (excluding the rectum), mutations were most common in the *SMAD4* gene. In tumors located on the right side, mutations were most common in *KRAS*, *PIK3CA*, *BRAF*, *MET*, and *PTEN*. Statistically significant differences in mutation frequency between the three primary tumor locations (left vs. rectum vs. right) were detected in the *TP53* (53.2% vs. 59.5% vs. 41.1%, p = 0.0083), *FBXW7* (5.2% vs. 10.5% vs. 2.7%, p = 0.0148), and *BRAF* (6.4% vs. 1.6% vs. 15.2%, p < 0.0001) genes. Similar to the findings in the right side vs. left side comparison, the *APC* gene showed similar mutation frequencies in the three tumor locations (Supplementary Table 7).

**Table 4:** Correlation between clinical features and gene mutations according to primary tumor location (left side, right side, and rectum).

	1	2	3	Total (N = 552)	p-value	p (1 vs. 2)	p (1 vs. 3)	p (2 vs. 3)
	Left (N = 250)	Right (N = 112)	Rectum (N = 190)					
Sex					0.0011	0.1055	0.01166	0.0003
Woman	100 (40.0%)	55 (49.1%)	54 (28.4%)	209 (37.9%)				
Man	150 (60.0%)	57 (50.9%)	136 (71.6%)	343 (62.1%)				
Age (years)					0.8594	0.6838	0.8396	0.5825
< = 49	21 (8.4%)	8 (7.1%)	17 (8.9%)	46 (8.3%)				
> = 50	229 (91.6%)	104 (92.9%)	173 (91.1%)	506 (91.7%)				
Age (years)					0.6304	0.3732	0.9895	0.3891
< = 64	113 (45.2%)	45 (40.2%)	86 (45.3%)	244 (44.2%)				
> = 65	137 (54.8%)	67 (59.8%)	104 (54.7%)	308 (55.8%)				
*BRAF*_all					< 0.0001	0.0073	0.0137	< 0.0001
No	234 (93.6%)	95 (84.8%)	187 (98.4%)	516 (93.5%)				
Yes	16 (6.4%)	17 (15.2%)	3 (1.6%)	36 (6.5%)				
*BRAF*_(only p.(Val600Glu))					< 0.0001	0.0074	0.0484	< 0.0001
No	241 (96.4%)	100 (89.3%)	189 (99.5%)	530 (96.0%)				
Yes	9 (3.6%)	12 (10.7%)	1 (0.5%)	22 (4.0%)				
*KRAS*_all					0.2258	0.1364	0.7087	0.0849
No	135 (54.0%)	51 (45.5%)	105 (55.3%)	291 (52.7%)				
Yes	115 (46.0%)	61 (54.5%)	85 (44.7%)	261 (47.3%)				
*NRAS*					0.6832	0.6542	0.5908	0.3992
No	236 (94.4%)	107 (95.5%)	177 (93.2%)	520 (94.2%)				
Yes	14 (5.6%)	5 (4.5%)	13 (6.8%)	32 (5.8%)				
Mutations					0.0006	0.01718	0.1467	< 0.0001
Without mutations BKN	107 (42.8%)	32 (28.6%)	90 (47.4%)	229 (41.5%)				
* BRAF*	14 (5.6%)	14 (12.5%)	3 (1.6%)	31 (5.6%)				
* KRAS*	115 (46.0%)	61 (54.5%)	84 (44.2%)	260 (47.1%)				
* NRAS*	14 (5.6%)	5 (4.5%)	13 (6.8%)	32 (5.8%)				
Tumor_recurrence					0.2150	0.3631	0.2812	0.0938
N-Miss	1	0	0	1				
No	229 (92.0%)	106 (94.6%)	169 (88.9%)	504 (91.5%)				
Yes	20 (8.0%)	6 (5.4%)	21 (11.1%)	47 (8.5%)				
ICD					NA			
C18	179 (71.6%)	112 (100.0%)	0 (0.0%)	291 (52.7%)				
C19	71 (28.4%)	0 (0.0%)	0 (0.0%)	71 (12.9%)				
C20	0 (0.0%)	0 (0.0%)	190 (100.0%)	190 (34.4%)				
Liver metastases					0.0311	0.01197	0.09505	0.2935
No	105 (42.0%)	63 (56.2%)	95 (50.0%)	263 (47.6%)				
Yes	145 (58.0%)	49 (43.8%)	95 (50.0%)	289 (52.4%)				
Lung metastases					0.0377	0.9543	0.0196	0.05836
No	198 (79.2%)	89 (79.5%)	132 (69.5%)	419 (75.9%)				
Yes	52 (20.8%)	23 (20.5%)	58 (30.5%)	133 (24.1%)				
Peritoneum metastases					0.0004	0.8929	0.0001	0.0002
No	220 (88.0%)	98 (87.5%)	186 (97.9%)	504 (91.3%)				
Yes	30 (12.0%)	14 (12.5%)	4 (2.1%)	48 (8.7%)				
Grade					0.0004	< 0.0001	0.1839	0.0330
N-Miss	12	5	11	28				
Adenoca G1	42 (17.6%)	15 (14.0%)	27 (15.1%)	84 (16.0%)				
Adenoca G2	185 (77.7%)	71 (66.4%)	136 (76.0%)	392 (74.8%)				
Adenoca G3	11 (4.6%)	21 (19.6%)	16 (8.9%)	48 (9.2%)				
T					0.0042	0.268	0.0407	0.0002
N-Miss	71	26	74	171				
T1	6 (3.4%)	0 (0.0%)	4 (3.4%)	10 (2.6%)				
T2	16 (8.9%)	7 (8.1%)	16 (13.8%)	39 (10.2%)				
T3	105 (58.7%)	45 (52.3%)	78 (67.2%)	228 (59.8%)				
T4	49 (27.4%)	33 (38.4%)	16 (13.8%)	98 (25.7%)				
Tis	2 (1.1%)	1 (1.2%)	0 (0.0%)	3 (0.8%)				
Tx	1 (0.6%)	0 (0.0%)	2 (1.7%)	3 (0.8%)				
N					0.1168	0.0508	0.4384	0.1998
N-Miss	71	26	74	171				
N0	70 (39.1%)	26 (30.2%)	36 (31.0%)	132 (34.6%)				
N1	65 (36.3%)	25 (29.1%)	46 (39.7%)	136 (35.7%)				
N2	42 (23.5%)	34 (39.5%)	31 (26.7%)	107 (28.1%)				
Nx	2 (1.1%)	1 (1.2%)	3 (2.6%)	6 (1.6%)				

*N-miss* number of cases without information, *ICD* International Classification of Diseases, *T* tumor, *N* node, *BKN BRAF, KRAS, NRAS*, *NA* not applicable.

Due to Bonferroni correction, p < 0.017 (p < 0.05/3) should be taken as statistically significant.

Gene mutations were more prevalent in men than in women in the three groups (right, left, and rectal) (Table 4), and the difference was significant between rectal cancer patients and those in the other groups (p = 0.0003, p = 0.011). *BRAF* gene mutations were more than twofold more frequent on the right than on the left side (15.2% vs. 6.4%) and significantly more frequent in right-sided tumors than in rectal tumors (15.2% vs. 1.6%, p < 0.0001). When the analysis of mutations in the *BRAF* gene was restricted to the p.(Val600Glu) mutation, the mutation frequency differed between right-sided tumors and left-sided tumors (10.7% vs. 3.6%, p < 0.0074), whereas rectal tumors (0.5%) differed greatly in frequency from left-sided tumors (0.5% vs. 3.6%; but after Bonferroni correction not significant p < 0.0484) and significantly from right-sided tumors (0.5% vs. 10.7%; p < 0.0001). For tumors located in the rectum, mutations in the *KRAS*, *NRAS*, and *BRAF* genes were absent in > 47.4% of patients compared with the left side (42.8%) and right side (28.6%) (p = 0.0006). Liver metastases were more frequent in patients with left-sided tumors than in those with rectal and right-sided tumors (58% vs. 50% vs. 43.8%) (p = 0.0311). Lung metastases were most frequent in patients with primary tumors in the rectum (30.5%) than in those with primary tumors on the left and right sides (20.8% and 20.5%) (p = 0.0377). Peritoneal metastases were more common in patients with right- (12.5%) and left-sided (12%) tumors than in those with rectal tumors (2.1%) (p = 0.0004). Tumors located on the right side most often showed the highest (19.6%) grade (G3) compared with tumors located in the rectum (8.9%) and on the left side (4.6%) (p = 0.0004). Similarly, right-sided tumors (more than 90%) were larger (T3-T4) than left-sided and rectal tumors (81%) (p = 0.0042). The remaining clinical features analyzed, such as age of onset (< 49 vs. > 50 and < 64 vs. > 65), *KRAS* and *NRAS* status, presence of recurrence, and lymph node metastasis (N) did not differ significantly between cases with tumors on the left side, right side, and rectum.

Generally, the majority of rectal tumor cases occurred in males, who were often suitable for anti-EGFR therapy, with 47.4% qualifying for this treatment. Lung metastases were most seen in cases of rectal tumors. For tumors situated on the left side, the liver and peritoneum were the usual metastatic sites, while right-sided tumors primarily spread to the peritoneum. Genetic analysis revealed a high association *of TP53* and *FBXW7* mutations in rectal tumors, as shown in Table 5. PIK3CA and BRAF mutations were rarer in rectal tumors compared to those on the right and left sides, as illustrated in Fig. 3B. Specifically, the *BRAF* p.(Val600Glu) mutation was exceptionally rare in rectal tumors, detected in only one out of 190 cases (0.5%). Furthermore, *TP53* gene mutations were more common in rectal tumors than in tumors of other locations, with occurrences of 59.5%, compared to 53.2% on the left side and 41.1% on the right side, as depicted in Fig. 3B.

**Table 5:** Tumor features according to tumor location: Right side, left side, and rectum.

	Right-sided	Left-sided	Rectum
Mutated genes	KRAS, PIK3CA, BRAF*, MET, PTEN	*SMAD4*	TP53*, FBXW7*, NRAS
Sex	Women = Men	Women < Men*	Women < Men*
*KRAS, NRAS, BRAF*; WT	28.6%*	42.8%*	47.4%*
Metastases	Peritoneum*	Liver*, Peritoneum*	Lung*
Tumor diameter	Bigger*	Smaller*	Smaller*
Grade	G3*	G2*	G2*

*Statistically significant, p < 0.05.

## Discussion

Among 552 CRC cases analyzed, mutations were most frequently detected in the following genes: *TP53*, *KRAS*, *APC*, *KDR*, *PIK3CA*, *SMAD4*, *BRAF*, *FBXW7*, *NRAS*, *MET*, and *PTEN*. Similar mutation frequencies were reported in a study that analyzed more than 400 genes in 1134 CRC cases, as well as in a study by El-Deiry et al. that included 6892 cases and a 45-gene panel^16,17^.

### Mutations in *TP53*

Mutations in the *TP53* gene were the most frequently detected in the cohort of 552 cases (Supplemental Tables 6 and 7). *TP53* mutations are associated with CRC progression and increased risk of metastasis^18^. Whether the presence of *TP53* mutations serves as a predictor of the response to chemotherapy remains unclear. Although the presence of TP53 mutations has been associated with a poor response of colorectal cancer to chemotherapy^19^, the predictive value of *TP53* mutations in advanced CRC patients treated with cytotoxic agents is not clear^20^. Additional studies are needed to assess the role of mutations in *TP53* on the increased number of neoantigens and thus on the response to immunotherapy^19^.

### Mutations in *KRAS*

*KRAS* was one of the most frequently mutated genes (47%, 261/552) among the 50 genes analyzed in the current study. Slightly lower frequencies (44%) were described by Yaeger and El-Deiry^16,17^. Although the mutational status of the *KRAS* and *NRAS* genes remains constant in primary tumors and in distant metastases^21^, the difference between the present data and previous reports may be due to the inclusion of tissue samples from metastases in previous studies^16,17^. A meta-analysis indicated that in 11.3% of cases with *KRAS* mutation in primary tumors, the mutation is not present in metastatic tissues. However, these results are derived from studies using molecular detection methods for *KRAS* with different sensitivities and analytical ranges^22^. Currently, researchers and clinicians are focusing on the *KRAS* p.(Gly12Cys) mutation, which is blocked by a specific small-molecule inhibitor (sotorasib or AMG510)^23^. We detected *KRAS* p.(Gly12Cys) mutations in 19 patients (3.4%). Although these patients are not eligible for anti-EGFR therapy, they are potential candidates for molecular targeted anti-KRAS therapy (e.g., AMG510). However, unlike the positive results reported in patients with NSCLC, AMG510 has not shown a clinical benefit in the treatment of CRC^24^. This may be due to the reactivation of EGFR. The first attempts to combine the AMG510 inhibitor with anti-EGFR therapy in a preclinical model showed promising results^25^. Recently, results of a clinical trial (NCT03785249) have been published that show the efficacy of a combination therapy of adagrasib (*KRAS* p.(Gly12Cys) small molecule inhibitor) with cetuximab (anti-EGFR monoclonal antibody) for the treatment highly pretreated metastatic CRC patients^26^. The *KRAS* p.(Gly12Cys) mutation, which is not an exclusion marker, is an indication for combination therapy (anti-KRAS + anti-EGFR). Studies assessing the effect of inhibitors of the mutant KRAS p.(Gly12Cys) protein have described several mechanisms involved in the acquisition of resistance to these drugs^27^.

### Mutations in *NRAS*

Mutations in *NRAS* were detected in 32 cases (5.8%). Similar frequencies were described previously (4–6%)^16,28^. In addition to the predictive value of *NRAS* mutations for anti-EGFR therapy, colorectal cancer patients with *NRAS* mutations have a shorter overall survival than those without^28^.

### Mutations in *BRAF*

There are three types of *BRAF* mutations^29^. Class I (affecting the Val600 codon) and class II (function as RAS-independent dimers) mutations do not require upstream activation and thus rarely co-occur with *RAS* mutations. The third type is class III (kinase impaired)^16^. In this study, *RAS* gene mutations were not detected in cases with a class I *BRAF* mutation (p.(Val600Glu) ). Val600 mutation inhibitors have been used in the treatment of cancer for several years, whereas lung and colorectal tumors with a class III *BRAF* mutation are sensitive to receptor tyrosine kinase inhibitors^30^. Universal inhibitors of *BRAF* class I/II/III mutations are currently under investigation, underscoring the clinical importance of the detection of the entire spectrum of mutations in the *BRAF* gene^31^.

### Mutations in *PIK3CA*

Mutations in the *PIK3CA* gene encoding the PIK3/AKT/MTOR pathway kinase, a second important pathway activated during carcinogenesis, correlate with resistance to anti-EGFR monoclonal antibodies^32^. This resistance is only found in patients with *PIK3CA* exon 20 mutations^33,34^. Prospectively, the incorporation of additional testing for *PIK3CA* exon 20 mutations in cases in which mutations in *KRAS*, *NRAS*, and *BRAF* are not detected could exclude an additional group of patients from unsuccessful anti-EGFR therapy. Future studies are necessary to determine whether the use of PI3K inhibitors would abolish the negative effect of *PIK3CA* mutations on the response to treatment^35,36^. The PIK/AKT pathway is frequently overactivated due to mutations in *PIK3CA* and comutation or repression of *PTEN*. In this study, the co-occurrence of *PIK3CA* and *PTEN* mutations was detected in seven (1.2%) of the analyzed cases. CRCs with the *PIK3CA* mutation and negative PTEN protein overexpress CD274 (PD-L1). PD-L1 protein is an immune checkpoint inhibitor targeting regulator^37^. In the cited study, PTEN protein expression was evaluated by immunohistochemistry, which detects the end result of various disorders of PTEN protein production. In this study, we only tested a nucleotide change in the gene encoding the PTEN protein.

### The p.(Gln472His) variant of the *KDR* gene

The *KDR* p.(Gln472His) variant was frequently detected in the present cohort. Despite the uncertain geminal significance of the *KDR* p.(Gln472His) variant in melanoma tissue, its presence is correlated with increased microvessel density compared with that in patients without the variant. Additionally, cell lines derived from patients with the variant show increased proliferation and a greater invasive potential and are more sensitive to targeted VEGFR2 inhibition than those without the variant^38^. Co-occurrence of the *KDR* p.(Gln472His) and *KIT* p.(Met541Leu) variants is associated with aggressive forms of glioblastoma multiforme^39^. The *KDR* p.(Gln472His) variant is listed as 'likely oncogenic' in the OncoKB database (https://www.oncokb.org/gene/KDR).

### Mutations in the *ERBB2* (*HER2*) gene

In addition to *ERBB2* gene amplification, mutations in the *ERBB2* gene have gained increased attention. In CRC, *ERBB2* mutations have been reported in 4% of cases in The Cancer Genome Atlas^40^. The p.(Leu755Ser) and p.(Val777Leu) mutations are considered oncogenic, whereas p.(Arg784His) and p.(Val842Ile) are considered likely oncogenic, according to the OncoKB database (https://www.oncokb.org/). These mutations are blocked by the inhibitors neratinib and afatinib. Detection of somatic mutations in the *ERBB2* gene has predictive potential for targeted therapies^41–43^. In this study, the panel used did not allow assessment of *HER2* gene amplification.

#### Left side vs. right side

CRC is not a homogeneous cancer, and data suggest that the development of this cancer is closely related to its localization (left vs. right side).

Consistent with previous data, the present results indicate that tumors located on the left side are more frequent among men and are associated with *TP53* gene mutations and liver metastases^15,16^. Cancers arising on the right side were associated with mutations in *BRAF*; they tend to be larger and frequently show a higher degree of malignancy at diagnosis^15,16^. The present analysis showed that 45% of patients with left-sided tumors did not have mutations in *KRAS*, *NRAS*, and *BRAF*; these patients are thus potential candidates for targeted therapies using anti-EGFR monoclonal antibodies. By contrast, only 28.6% of cancers located on the right side were eligible for anti-EGFR therapy. Recent meta-analyses indicate that *RAS*-WT patients with tumors located on the left side have better treatment outcomes than those with tumors on the right side, including patients after anti-EGFR monoclonal antibody therapy^44,45^. This is reflected in the current ASCO recommendations, that anty-EGRF therapy is not recommended as first-line therapy for patients with right-sided *RAS-*WT metastatic tumors^46^.

#### Left side vs. right side vs. rectum

In this study, rectal tumors were considered separately from left-sided tumors because the clinical management of cancers in the rectum differs from that of other CRCs^8^. Patients with rectal tumors were predominantly male and potential candidates for anti-EGFR therapy (47.4%). The most common site of metastasis from rectum-localized tumors was the lung, whereas tumors located on the left side metastasize to the liver and peritoneum, and those on the right side metastasize to the peritoneum. Mutations in the *TP53* and *FBXW7* genes were frequently associated with tumors located in the rectum^16,47^. In this study, *KRAS* mutations were less frequent in tumors located in the rectum than in right-sided and left-sided tumors, which is not consistent with previous findings^16,47^. Recently published results from the prospective phase II trial (EXCITE), which included 76 rectal cancer patients who underwent R0 resection, indicate high heterogeneity according to the presence of *KRAS* and *TP53* mutations in the comparison of biopsy samples obtained before neoadjuvant treatment (chemoradiation) and after treatment (resection material). The researchers also noted that *RAS*-WT tumors more often showed excellent clinical or pathological response^48^. In this study, *PIK3CA* and *BRAF* mutations were less frequent in the rectum than in the other primary tumor locations (right and left sides). Similar results were reported by El-Deiry et al. (rectal cancer, 10% *PIK3CA* and 3% *BRAF*)^17^. Consistent with previous studies, mutations in the *BRAF* gene occurred at a low frequency in rectal tumors, and the *BRAF* p.(Val600Glu) mutation was present in only one case (1/190, 0.5%)^49^.

Mutations in the *TP53* gene were more frequent in rectal tumors than in tumors in other locations (left side and right side) (59.5% vs. 53.2% vs. 41.1%). The high frequency of *TP53* mutations in tumors located in the rectum was reported previously (81%)^16^. The role of *TP53* status in the response to radiotherapy in rectal cancers has been studied extensively. However, the relationship between *TP53* status and the response of rectal cancer to neoadjuvant radiotherapy is inconsistent^50,51^. The predictive significance of *TP53* status may also depend on the type of mutation, such as loss-of-function, dominant-negative effect, or gain-of-function mutations^52^. In addition to mutations in *TP53*, mutations in the *FBXW7* gene were significantly more common in the rectum than in left- and right-sided tumors (10.5% vs. 5.2% vs. 2.7%). Similar frequencies of *FBXW7* mutations in rectal tumors were reported previously (9.9%)^53^. The *FBXW7* gene encodes a tumor suppressor protein involved in the ubiquitin-mediated proteasomal degradation of several oncoproteins (cyclin E, c-Myc, Mcl-1, mTOR, Jun, Notch, and AURKA)^54^. Mutations in the *FBXW7* and *SMAD* genes are more common in patients who are resistant to anti-EGFR immunotherapy (cetuximab or panitumumab)^55^. As reported previously, low *FBXW7* expression in tumors is associated with a poor prognosis^56^, and loss of the *FBXW7* gene correlates with resistance to oxaliplatin, which is often used to treat disseminated CRC^57^.

The strength of this study was the inclusion of a large cohort of patients who were mostly surgically treated and diagnosed at a single institution, as well as the use of NGS as a routine diagnostic method for stratifying patients for targeted therapies. The study had some limitations in addition to its retrospective design. We were not able to assess overall survival. Survival time could not be assessed because follow-up is ongoing, and further studies are thus warranted. MSI/MMR status was not assessed using molecular biology/immunohistochemistry methods because this was not the standard procedure in our institution at that time.

## Conclusions

The results of this retrospective analysis indicate that tumor location has an impact on the potential treatment of CRC with anti-EGFR therapy. Tumors located in the rectum showed differences in biology, metastatic rate (lung), and mutation frequency (e.g., *BRAF, FBXW7*, and *TP53*) from those in other locations (left side and right side). The location of the primary tumor and the mutation status of *KRAS, NRAS*, and *BRAF* showed greater prognostic and predictive value than the current molecular classifications of CRC.

## Material and methods

This retrospective analysis used data from CRC molecular diagnostics performed between 2016 and 2020. A total of 552 cases of CRC diagnosed at Holy Cross Cancer Center by NGS were included in the study (Table 1). The clinical data were anonymized. The subjects included 209 (38%) women and 343 (62%) men. The mean age at diagnosis was 64 (SD 10) (range: 27–90) years, and the median age was 66 years (IQR: 59–71). There were 388 (70%) patients with stage IV cancer and 82 (15%) with stage III cancer. The remaining 15% were patients in clinical stages 0–II. The primary tumor was located on the left side in 440 (80%) patients, including 190 (34%) patients with tumors in the rectum and 250 (45%) patients with tumors on the left side; there were 112 patients (20%) with tumors on the right side. Histopathologically, 98.9% of cases were adenocarcinoma, and there was one case each of goblet cell carcinoid (0.2%), medullary carcinoma (0.2%), and neuroendocrine carcinoma (0.2%). In three cases (0.5%), data on the diagnosis were not available (Table 1).

### DNA isolation

A pathologist marked the area containing tumor cells on a hematoxylin- and eosin-stained slide. The tumor cell content of the marked area varied between 10 and 100% in all cases, and the median was 70%. The tumor tissue on matched unstained slides was deparaffinized, and the selected area was transferred to a tube for DNA isolation using the Maxwell 16 and Maxwell® 16 FFPE Tissue LEV DNA Purification kits according to the manufacturer’s instructions (Promega, USA). The concentration of the isolated DNA was measured using Qubit 2.0 (Thermo-Fisher Scientific). The mean concentration of isolated DNA was 100 ng/µl, and the mean purity measured by the 260/280 ratio was 1.8–2.0.

### Next generation sequencing

#### Library preparation

DNA was diluted to 10 ng/µl. Libraries were prepared using the Ion AmpliSeq™ Cancer Hotspot Panel v2 Kit (Thermo-Fisher Scientific) and the Ion Xpress Barcode Adapters Kit (Thermo-Fisher Scientific), according to the manufacturer’s instructions. The Ion AmpliSeq™ Cancer Hotspot Panel v2 Kit allows the study of 50 tumor-related genes (*ABL1*, *EZH2*, *JAK3*, *PTEN*, *ACT1*, *FBXW7*, *IDH2*, *PTPN11*, *ALK*, *FGFR1*, *KDR*, *RB1*, *APC*, *FGFR2*, *KIT*, *RET*, *ATM*, *FGFR3*, *KRAS*, *SMAD4*, *BRAF*, *FLT3*, *MET*, *SMARCB1*, *CDH1*, *GNA11*, *MLH1*, *SMO*, *CDKN2A*, *GNAS*, *MPL*, *SRC*, *CSF1R*, *GNAQ*, *NOTCH1*, *STK11*, *CTNNB1*, *HNF1A*, *NPM1*, *TP53*, *EGFR*, *HRAS*, *NRAS*, *VHL*, *ERBB2*, *IDH1*, *PDGFR*, *ERBB4*, *JAK2*, and *PIK3CA*). This panel includes tumor suppressor genes and oncogenes in which mutations occur most frequently in cancer.

The resulting multiplex PCR products were subjected to partial enzymatic digestion to remove primer sequences. Then, adapters for multiplex PCR products were enzymatically attached using the Ion Xpress Barcode Adapters Kit (Thermo-Fisher Scientific). Each adapter contains barcodes that allow identification of sequences from a given patient among a mixture of libraries. The prepared libraries were cleaned using Agencourt AMPure XP (Beckman Coulter Genomics) according to the manufacturer’s instructions (Ion AmpliSeq Library Kit 2.0, Thermo-Fisher Scientific).

#### Preparation of clonally amplified templates for sequencing: Emulsion PCR (emPCR) for S5 using IonChef

The concentration of libraries was measured by quantitative PCR with real-time detection (qRT-PCR) using the Ion Library TaqMan™ Quantitation Kit (Thermo-Fisher Scientific) on a Rotor-Gene Q instrument (Qiagen). According to the values obtained by qRT-PCR, the prepared libraries were diluted to a concentration of 100 pM. Then, emPCR was performed using Ion Chef (Thermo-Fisher Scientific) and Ion 520 & Ion 530 Kit-Chef and Ion 530™ Chip Kit (Thermo-Fisher Scientific). After enrichment, two 530 chips were loaded (16–24 samples per chip, sequencing depth × 500).

### Sequencing

Sequencing was performed on an Ion S5 sequencer (Thermo-Fisher Scientific) according to the manufacturer’s instructions.

### Bioinformatic analysis

The raw data generated by sequencing were processed using the Torrent Server Suite (TSS) 5.12 (Thermo-Scientific, USA). The obtained sequences were matched (mapped) to the reference sequence of the human genome (hg19). A search for different variants (SNP, deletions, insertions) was performed using the Variant Caller 5.12 program, which is part of TSS 5.12. The following basic parameters of the variants were used: minimum allele frequency, SNP = 0.01/INDEL = 0.05; minimal quality, 10; and minimal sequencing depth, 10. Variant Caller is compatible with the IGV genomic browser, Integrative Genomics Viewer (Broad Institute), which enables fast visualization of sophisticated variants. The detected variants were annotated with TSS using the wANNOVAR software (www.wannovar.usc.edu). Additionally, TSS 5.6-generated FASTQ files were used for analysis using the CLC Biomedical Workbench 5.0 (QIAGEN). The basic parameters used for CLC were as follows: minimum allele frequency, 0.01; minimal quality, 10; and minimal sequencing depth, 100. Detected mutations, SNPs, insertions, and deletions of the coding regions of the analyzed genes were filtered to detect pathogenic mutations using the COSMIC database, dbSNP database (to discard hereditary polymorphisms), and the population database of the 1000GENOMES project. Only variants with a minimal allelic frequency of 5% were reported.

### Classification of mutations

Detected mutations were classified according to the information deposited in the ClinVar database. For variants of unknown significance or conflicting results, in silico analysis was performed using Varsome (https://varsome.com/), which integrates useful algorithms, databases, population frequencies, and literature citations. Figures were produced using the maftools package and R and RStudio^58–60^.

### Statistical analysis

Categorical data were summarized by frequencies and percentages and compared using the chi-square or Fisher’s exact test. A two tailed p-value < 0.05 was considered statistically significant. Bonferroni correction (p < 0.05/3; p < 0.017) was applied to the results in Table 3. All statistical analyses were performed using R version 4.3.0^59^.

### Ethics declarations

This study was conducted in accordance with the Declaration of Helsinki and approved by the Institutional Review Board at the Holycross Chamber of Physicians in Kielce (10/2016). The whole clinical and molecular data were anonymized. Informed consent was obtained from all subjects involved in the study.

### Supplementary Information

Supplementary Tables.

Supplementary Figure 1.

## Data Availability

All data generated and/or analyzed during this study are included in this manuscript and its supplementary information files.
