# Supplementary material for: Tumor location matters, next generation sequencing mutation profiling of left-sided, rectal, and right-sided colorectal tumors in 552 patients
Source: Sci Rep. 2024 Feb 26;14:4619. doi: 10.1038/s41598-024-55139-w (PMC10897470; doi:10.1038/s41598-024-55139-w)
Supplement: Supplementary file 2 — Supplementary Figure 1. [file 41598_2024_55139_MOESM2_ESM.pdf]

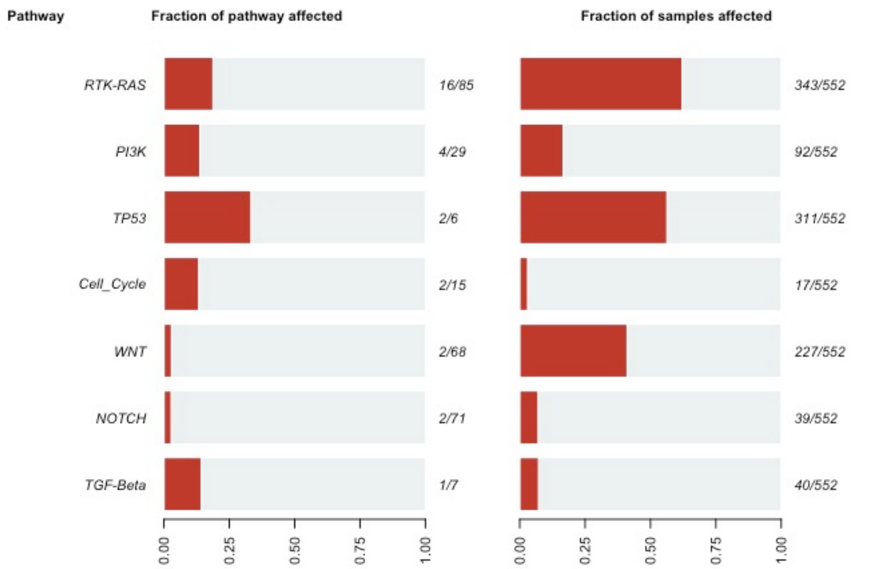

Supplementary Figure 1. The fraction pathway and samples affected based on identified mutations in whole study group of 552 cases of CRC.
